# The Legacy of Harrington’s Rod and the Evolution of Long-Segment Constructs in Spine Surgery

**DOI:** 10.3390/jcm13185556

**Published:** 2024-09-19

**Authors:** Iheanyi J. Amadi, Jean-Luc K. Kabangu, Adip G. Bhargav, Paul J. Camarata

**Affiliations:** 1 School of Medicine, University of Kansas, Kansas City, KS 66160, USA; 2 Department of Neurological Surgery, University of Kansas Medical Center, Kansas City, KS 66160, USA

**Keywords:** Harrington rod, spinal surgery evolution, scoliosis treatment, spinal deformities, neurosurgical innovations, segmental pedicle screws

## Abstract

This paper delves into the historical evolution of spinal surgery, focusing on the pivotal role of the Harrington rod in treating spinal deformities. Introduced in 1955, the Harrington rod marked a significant breakthrough in neurosurgery, especially for scoliosis treatment, by offering a novel approach to spinal stabilization. Through a retrospective analysis, this study examines the development and impact of the Harrington rod, highlighting Dr. Paul Harrington’s contributions to spinal surgery. His innovative technique revolutionized the management of spinal deformities, laying the groundwork for future advancements in spinal instrumentation. Despite initial skepticism, Harrington’s methods gained acceptance, significantly influencing neurosurgical practices and patient outcomes. This study also explores subsequent advancements that built on Harrington’s work, including the transition to long-segment spine constructs and the introduction of segmental pedicle screws, which allowed for more precise deformity correction. Reflecting on Harrington’s legacy, this paper acknowledges the continuous evolution of spinal surgery, driven by the interplay between clinical challenges and technological innovations. Harrington’s pioneering spirit exemplifies the ongoing pursuit of better surgical outcomes, underscoring the importance of innovation in the field of neurosurgery.

## 1. Introduction

The turn of the 20th century witnessed increasing interest in the treatment of spinal deformities associated with tuberculous spondylitis or Pott’s disease and poliomyelitis. Severe cases of spinal deformities and scoliosis, which result in poor posture, were often believed to lead to considerable morbidity and mortality at that time [1,2,3]. The imperative to effectively correct and manage these conditions prompted the exploration and development of various interventions [4].

Among these interventions, Fritz Lange’s contribution to internal fixation in spine surgery is particularly noteworthy. Drawing inspiration from the use of splints in long bone fractures, Lange adapted this concept to the treatment of spinal deformities [5,6]. In 1908, he introduced an innovative technique to stabilize deformed or scoliotic spines using steel wires [5,6]. These 5 mm thick wires were placed on either side of the spinous processes and secured with silver wires. Although the method successfully slowed the progression of spinal deformities, it was not without challenges, as patients experienced infections and irritation from the wires. To address these complications, Lange later refined his technique by using tin-coated steel wires and silk for fixation, which led to improved patient outcomes [5]. Despite his focus on preventing deformity progression rather than correcting it, Lange’s pioneering work laid the foundation for future advancements in spinal surgery.

The Hibbs method for spinal arthrodesis, introduced in 1911, is also worth highlighting. This method involved mobilizing the spinous processes and bridging the interspinous gap using autologous bone grafts [7,8]. While revolutionary at the time, its outcomes were marred by challenges such as the loss of curvature correction and suboptimal recovery [9]. Consequently, plaster casts were used to provide preliminary stabilization and offer rigid post-operative support during spinal fusion, requiring patients to endure cumbersome bracing for up to nine months [10]. The next three decades saw various attempts at slowing the progression of scoliosis without the use of instrumentation up to the development of the “Milwaukee Brace” and Risser casts in the early 1950s [7,11,12,13].

The 1960s marked a transformative era in spinal surgery. There was a paradigm shift in the medical community towards the use of surgical instrumentation to enhance biomechanical stability and facilitate spinal fusion [13]. A landmark development of this era was the introduction of the Harrington rod in 1955 by Paul Harrington. This innovation, specifically designed for the correction and stabilization of neuromuscular scoliosis, revolutionized spinal surgery. Harrington’s invention addressed a critical gap in treatment options, significantly altering the field and leaving a lasting impact. This paper aims to delve into Harrington’s contributions, arguing that the Harrington rod was not merely a major milestone in scoliosis treatment but also a foundation for future advancements in spinal instrumentation. We explore how Harrington’s ingenuity and pioneering work fundamentally shaped the progression of spine surgery.

This comprehensive historical vignette delves into the life and groundbreaking work of Dr. Paul Harrington, a true pioneer in the field of spine surgery. At its core, the narrative explores the inception and design of the Harrington rod, a seminal contribution that revolutionized the landscape of spinal deformity management. Drawing upon a rich tapestry of primary historical archives, clinical research findings, and biomechanical studies spanning an impressive six decades, this study serves as a valuable repository of knowledge. It provides a nuanced and insightful historical perspective on contemporary spine instrumentation, shedding light on the challenges, triumphs, and transformative moments that defined an era in spine surgery.

## 2. Background of Dr. Paul Harrington

### 2.1. Early Life

Paul Randall Harrington was born on 27 September 1911, in Kansas City, Kansas [14]. He was a true product of his hometown, having passed through the Kansas City public school system. At age 6, he started his educational journey at Riverview School as a first grader. From September 1922, he attended Prescott School for fifth and sixth grade, where his scholastic and athletic ability began to manifest more prominently. Excelling in subjects such as language and arithmetic, Harrington also demonstrated a flair for extracurricular activities, including music and sports [14]. His early participation in the grade school basketball team hinted at his budding athletic talent.

In September 1924, he transitioned to Kansas City, KS Central Junior High School. These formative years were a precursor to a remarkable career characterized by persistence, leadership, and creative ingenuity. He was elected president of his class in the seventh grade and also assumed the role of president of the student council in ninth grade, a testament to his natural leadership ability [15]. His outstanding scholarship earned him a place in the National Honor Society during his junior year [16].

A glimpse into Harrington’s creative ingenuity emerged in his ninth-grade project, a yearly endeavor where students selected a project to work on independently throughout the school year. For his project, he constructed a model racing yacht, showcasing the intricate points of differentiation between a yacht and other boats [17]. This early display of craftsmanship and interest in mechanics will culminate in him building his boat, the Twin Star, during his early years as a physician [18] and, ultimately, the design of the Harrington rod and internal spinal fixation technique.

**Figure 1:**
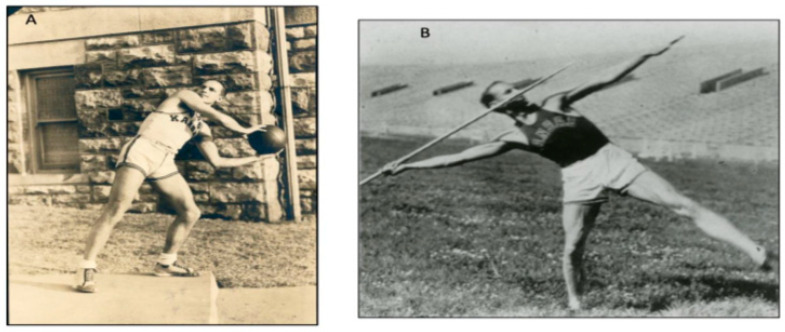
Photographs of Paul Harrington as a student–athlete at the University of Kansas. (**A**) A photograph showing Harrington during the official team photo for the basketball team c. 1933. (**B**) Harrington at a javelin meet c. 1934 (Used with permission from the Harrington Archives, Department of History and Philosophy of Medicine, University of Kansas Medical Center).

Harrington’s high school journey was not confined to academics alone; he thrived in sports, serving as the captain of the basketball team, and a member of the track team. He also played on the tennis doubles and baseball teams. He would go on to win state championships, medals, and various accolades during this time. Beyond the realm of sports, Harrington also excelled in the performing arts, showcasing his musical talent as a trumpeter in the band and taking on lead roles in plays presented by the opera/drama club of his school [19]. Harrington pursued higher education at the University of Kansas on a basketball and track scholarship from 1930 to 1934 (Figure 1). He was an integral member of the dominant KU basketball team that won the Big Six championship for three consecutive years, serving as the captain in 1934.

### 2.2. Medical Education and Military Career

Harrington transitioned to the University of Kansas School of Medicine in the fall of 1934, completing the first two years of his medical education at the University of Kansas campus in Lawrence. He moved back to the University of Kansas Medical Center in Kansas City, Kansas, for his clinical years and graduated in 1938. Harrington played semi-professional basketball during his days as a medical student to support his education.

**Figure 2:**
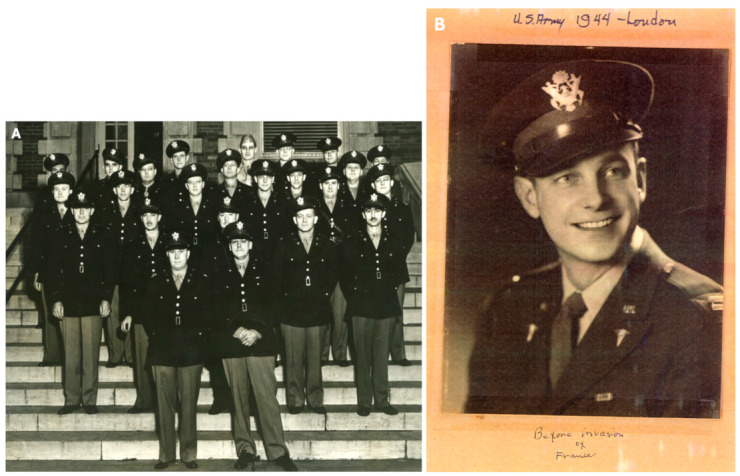
Pictures of Harrington during his military service. (**A**) Members of the 77th Evacuation Hospital Unit. Capt. Harrington, 3rd row, fourth from the right. (**B**) A portrait of Harrington in military attire, dated 1944. A handwritten text that reads “Before invasion of France” can be seen in this picture. (Used with permission from the Harrington Archives, Department of History and Philosophy of Medicine, University of Kansas Medical Center).

Harrington harbored a longing to serve in the military from an early age, expressing his interest in joining the US Navy in 1929, despite being too young for enlistment [20]. After graduating from medical school, Harrington completed an internship at Roper Hospital in Charleston, South Carolina, before returning to Kansas City, Missouri, for his orthopedic residency in the Dickson-Diveley Clinic at St. Luke’s Hospital. His aspiration to serve in the military materialized during World War II, coinciding with his residency. Joining the Medical Corps, he became a member of the 77th Evacuation Hospital, predominantly staffed by medical professionals from the University of Kansas Medical Center and the Kansas City area (Figure 2). Harrington was a part of multiple tours of the 77th Evac Hospital to Europe and North Africa, rising to the rank of Major by the time the war ended [21].

It is plausible to assert that Paul Harrington’s background in athletics and the military played a substantial role in shaping his later career innovations and fostering his creative acumen. As an athlete and team captain, he likely appreciated the importance of collaboration, diligence, and teamwork—values honed through a combination of grit, hard work, and talent. It is little wonder he was often an impactful part of successful teams as an athlete and also built successful collaborations with other professionals and organizations as highlighted in the next section.

Furthermore, Harrington’s capacity for critical thinking and creative insight was evident in both his military service and professional endeavors. His ability to question established methods and procedures was evident during the war, as exemplified in a February 1944 letter addressed to Colonel Rex Dively, a Senior Consultant in orthopedic surgery with the United States Army. In this letter, Harrington advocated against adopting the Tobrouk splint—a prevalent European technique then embraced by the US Army [22]. These qualities, essential in competitive team sports, played a crucial role in shaping Harrington’s multifaceted career.

## 3. The Inception of the Harrington Rod

Following the completion of his orthopedic surgery residency, Paul Harrington relocated to Houston and secured a position at the Jefferson Davis Hospital, where he developed an interest in patients with poliomyelitis. Houston had some of the highest infection rates during the poliomyelitis epidemic of the post-World War II years. As such, Harrington found himself in the polio unit that was suddenly overwhelmed with children at significant risk or already suffering from thoracic insufficiency syndrome secondary to poliomyelitis.

Harrington himself would acknowledge that the polio epidemic of the early post-World War II period provided the necessary impetus for the creative ingenuity that culminated in the development of the Harrington rod [14]. Existing techniques including those introduced by Hibbs and Ablee were no longer adequate for the management of neuromuscular scoliosis from polio [9,23]. Moreover, they were particularly not suitable for the pediatric population [20]. It is worth noting that Harrington’s interest in scoliosis was twofold. First, he sought to understand the etiology and pathophysiology of adolescent idiopathic scoliosis [24,25]. Secondly, he was also intrigued by the biomechanics of the trajectory of spinal deformity in patients with polio and neuromuscular scoliosis [26]. Beyond that, he was motivated to find a lasting approach for the correction of scoliotic spinal deformities [27].

**Figure 3:**
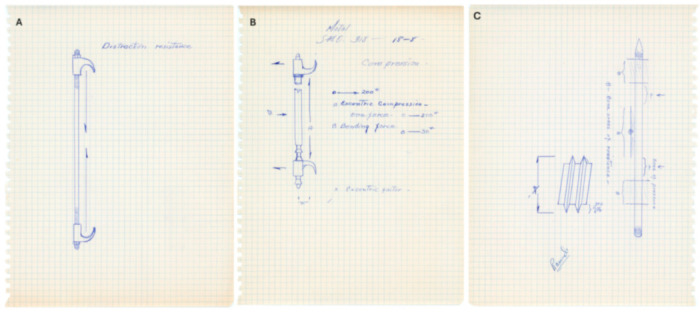
Some of the earliest free-hand sketches of Harrington instrumentation during the conception, design and experimental phases c. 1955. (**A**) Sketch of a distraction resistance rod and hook system (**B**) Appears to be a rod and hook system for compression resistance (**C**) Another sketch an early design of the Harrington Instrumentation. This might be a sacral bar or a version of a rod (Used with permission from the Harrington Archives, Department of History and Philosophy of Medicine, University of Kansas Medical Center).

Harrington spent the early 1950s seeking to identify and understand the mechanics of spinal instability. His ultimate goal was to develop an approach that restores spinal stability without the major shortcomings of previous techniques. Focusing mainly on the pediatric population, he discovered that a defect in the stability of the spine, as seen in scoliosis, results in severe deformities “directly related to the amount of instability and the duration of growth potential remaining in the patient” [27]. He observed that young patients with scoliosis had curvatures that progressed to exceed 100 degrees. As such, correcting the deformity while the patients were younger was crucial, their deformities being milder and physiologically more tolerable. Harrington sought to identify the forces required to correct or stop the progression of spinal curvature, envisaging that an effective application of relevant mechanical forces can re-establish spinal stability in scoliosis patients without negatively affecting vital functions [27]. Thus, he saw this as a biomechanical problem that required a biomechanical solution beyond available spinal interventions at the time. Figure 3 shows some of Harrington’s earliest free-hand sketches of the spinal instrumentation he was conceptualizing.

**Figure 4:**
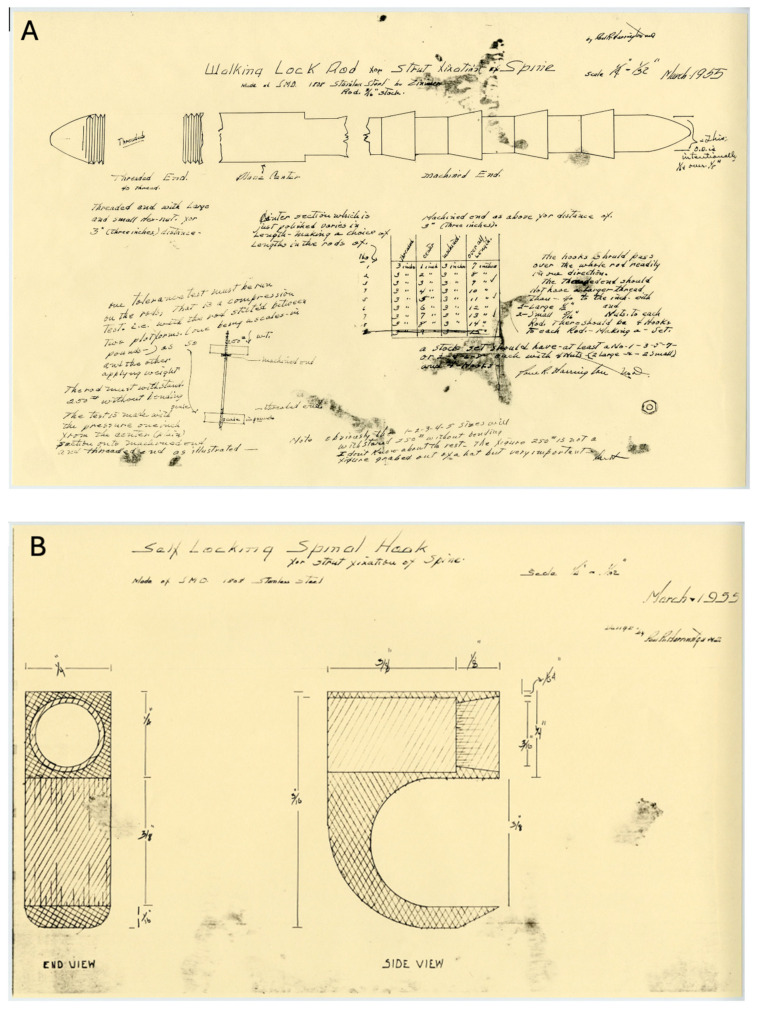
Biomechanical drawings of Harrington instrumentation c. 1955. (**A**) Walking lock rod for strut fixation. (**B**) Self-locking spinal hook (Used with permission from the Harrington Archives, Department of History and Philosophy of Medicine, University of Kansas Medical Center).

The Harrington instrumentation was borne as a possible solution to this biomechanical problem. It is based on a fundamental principle: spinal stabilization by applying a distraction force and a compression force to the posterior elements of the vertebrae in a synergistic manner using internal fixation (Figure 4). Stainless steel rods offer a tough support that can provide adequate distraction and compression forces to achieve correction, stabilization, adjustment, and fixation [28]. There was the question of whether the human body can tolerate metallic implants. However, Harrington acknowledged that stainless steel was not completely inert in the human body, but demonstrated that it was reasonably tolerated in most patients he had treated with these implants [27]. Hence, this approach addresses the need for a robust support system capable of maintaining spinal alignment and stability, going beyond merely stopping the progression of spinal deformities to actually correcting them.

**Figure 5:**
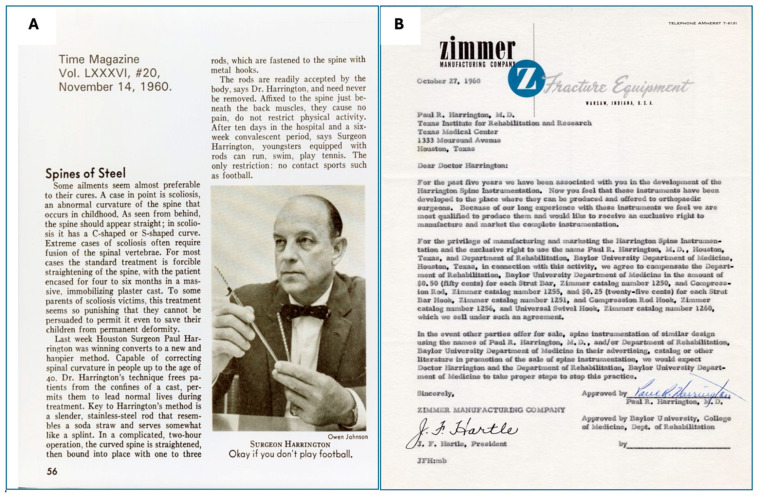
(**A**) Time magazine feature article on Harrington rod and technique. (**B**) Contract letter from Zimmer Inc., Warsaw, Indiana, USA (Used with permission from the Harrington Archives, Department of History and Philosophy of Medicine, University of Kansas Medical Center).

By 1955, Harrington conceived the idea of a hook and ratchet mechanism on a rod (Figure 5): the “Walking Lock Rod for Strut Fixation of Spine and Self Locking Spinal Hook.” [14]. The surgical technique would involve aligning the spine correctly and then stabilizing the convex facet joints using transfacet screws. This helped secure the spine in its new alignment. Careful patient selection was an integral aspect of Harrington’s use of instrumentation for the treatment of scoliosis. He developed a formula—degrees of curvature divided by the number of vertebrae in the scoliotic curve—to determine when to use instrumentation. Patients with a factor of less than 3 are managed conservatively, and patients with a factor of 5 are optimal candidates for instrumentation [27].

Harrington’s earlier techniques involved instrumentation with no plans for fusion. However, he quickly found that “Fusion of the spine is mandatory in conjunction with spine instrumentation to ensure long-term results” [29]. He recognized that several factors likely contribute to ultimately ensuring spinal fusion after instrumentation, arguing that appropriate post-operative management was essential in preventing pseudoarthrosis [27]. Thus, Harrington believed that instrumentation with fusion significantly improved the chances of maintaining spinal correction over time.

The development of the Harrington instrumentation system was significantly enhanced through fruitful collaborations with various professionals and organizations. In the 1950s, Thorkild Engen, an orthotist and engineer from Warm Springs, Georgia, provided invaluable expertise in refining the instrumentation system [14]. Engen’s specialized knowledge and his collaborative relationship with Harrington greatly contributed to the advancement in the new system. Additionally, Harrington tested and developed some of his original ideas with the Engineering Department at Rice University [30]. This shows Harrington’s willingness to collaborate and take advantage of opportunities and resources that allowed for the rigorous evaluation and refinement of his instrumentation system.

Similarly, Zimmer Incorporated, a prominent orthopedic equipment manufacturer based in Warsaw, Indiana, also played a vital role in the success of the Harrington instrumentation system [14,30]. Founded by Justin O. Zimmer in 1926, Zimmer Inc. had established itself as a leader in orthopedic equipment. The partnership between Harrington and Zimmer facilitated the production and distribution of the new spinal instrumentation. Zimmer’s manufacturing capabilities and industry experience were instrumental in bringing the innovative design to market. These successful collaborations and partnerships led to transformative advancements in Harrington’s spinal instrumentation system.

## 4. Clinical Implementation and Impact

Harrington was constantly refining his techniques and instruments to optimize outcomes [27,31]. The polio epidemic of the late 1940s highlighted the problem of scoliosis, particularly in patients with significant trunk involvement and cardiopulmonary issues. After Harrington moved to Houston to take the position at Baylor, he was at the forefront of this crisis [14,32]. At the time, there were no effective treatments for scoliosis. This motivated Harrington to find a solution for this devastating condition [32].

In 1954, a grant from the National Foundation for Infantile Paralysis was crucial in supporting Harrington’s early research and the development of his innovative instrumentation [19,32]. Harrington documented his patients’ post-operative progress, using this data to modify and improve his system. At the 1961 AOS conference, he reported on 172 cases of patients who had undergone his procedure and instrumentation over the past decade. These cases were instrumental in testing and improving various aspects and stages of development of his system, including the design of the instrumentation, physiological responses, adaptability, and reproducibility at different medical centers [28].

Harrington also published case series demonstrating the outcomes of patients treated with the Harrington rod. His studies tracked various outcomes, such as curvature reduction, curve progression arrest in children, functional improvement, and decreases in respiratory distress, fatigue, and pain, as well as the psychosocial impact of deformity [31]. In a study of 129 patients from a pool of 3000 polio sufferers over a decade, Harrington divided the patients into three groups based on when they received the instrumentation [31]. The first group received the original instrumentation, while the second and third groups were treated with progressively improved versions. Harrington observed significant incremental improvements in outcomes with each subsequent group [31]. It is important to highlight that the patients in this series were likely included in the 172 cases he reported at the AOS conference in 1961.

Harrington’s pioneering methods faced initial rejection and skepticism, particularly in the later half of the 1950s. However, a significant shift occurred in the early 1960s. In 1960, Harrington’s application to the American Academy of Orthopedic Surgeons (AAOS) was finally accepted, marking a crucial milestone. Influential figures in orthopedic surgery, such as John Moe, William Fisher, and Jim Hite, started to take a keen interest in Harrington’s instruments, engaging in meaningful exchanges of ideas with him. Moe was one of the first to adopt the Harrington instrumentation in the 1950s and is credited to have convinced Harrington to incorporate fusion as an ultimate goal of spinal fixation [2,32,33].

A pivotal moment came with Harrington’s 1960 thesis submission to the American Orthopedic Association (AOA), titled “Surgical Instrumentation for Management of Scoliosis.” This thesis is widely considered a major turning point in the history of Harrington’s spine instrumentation. Between 1960 and 1962, Harrington’s instrumentation and techniques gained increasing acceptance. He traveled to major cities and medical centers in the U.S. including Boston, New York, and Minnesota, where he operated on patients and imparted his knowledge. By 1965, Harrington’s influence had extended globally, with travels to Europe, Australia, and South America. He took on the role of a mentor, hosting surgeons at his hospital who sought to learn his groundbreaking technique. Notably, Harrington remained dedicated to refinement, constantly modifying and updating his instruments and techniques to address emerging challenges.

**Figure 6:**
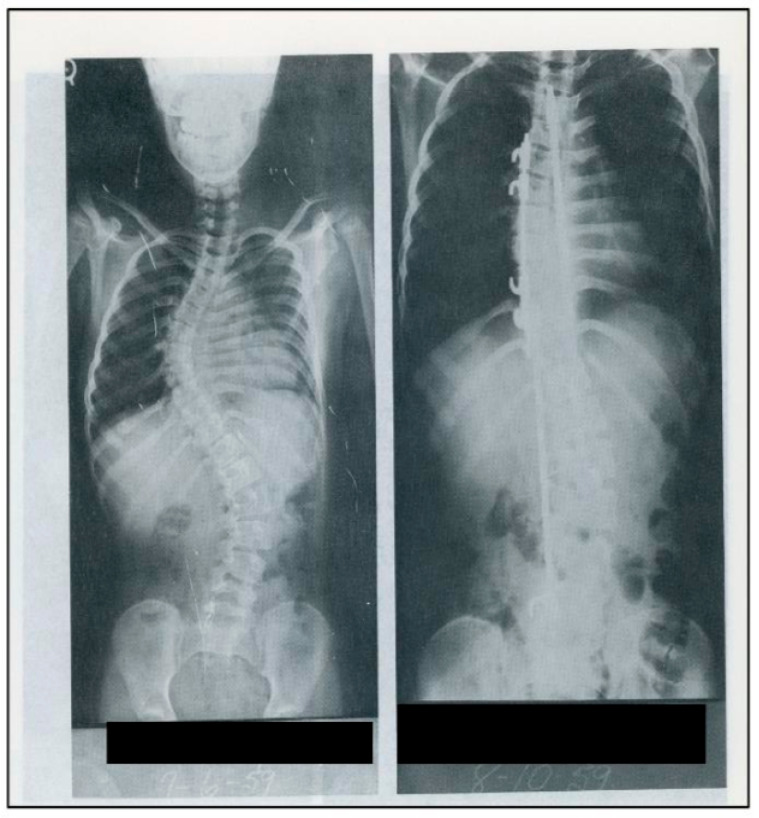
Pre- and post-operative radiographs of a 12-year-old scoliotic patient of Harrington’s after internal fixation with Harrington instrumentation, 1959 (Used with permission from the Harrington Archives, Department of History and Philosophy of Medicine, University of Kansas Medical Center).

A feature article in Time magazine on 14 November 1962 brought Harrington’s technique and instrumentation into the public limelight (Figure 6). This exposure transcended medical and academic circles, leading to an influx of letters from scoliosis patients and their families seeking guidance in making decisions about various medical and surgical interventions for scoliosis treatment [34].

**Figure 7:**
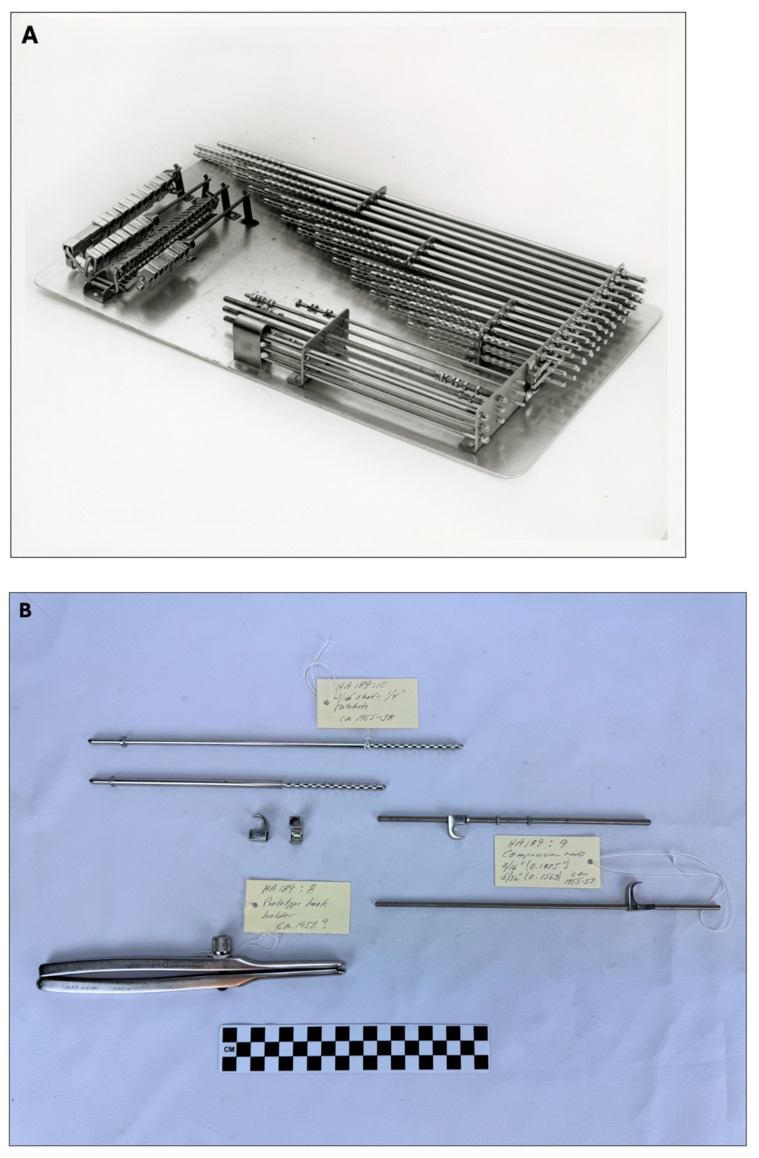
Pictures of Harrington instrumentation. (**A**) Complete set of Harrington instrumentation. (**B**) Pictures of prototype hook holder, compression rods, and ratchets (Used with permission from the Harrington Archives, Department of History and Philosophy of Medicine, University of Kansas Medical Center).

This widespread interest and acknowledgment from the public underscored the transformative impact of Harrington’s contributions in the field of spine surgery. The Harrington rod and instrumentation would become the standard of care for the management of scoliosis until the early 1980s [7] and patient satisfaction remained high several years after instrumentation [35]. Figure 7 shows a complete set of the prototype of Harrington instrumentation.

## 5. Limitations and Complications

A notable drawback of the Harrington rod is the loss of normal spine curvature. The distraction and compression forces cause muscles to work at a biomechanical disadvantage [36]. This ultimately led to the flattening of the spine in both the sagittal and coronal planes. This phenomenon gives rise to what is now recognized as the flatback syndrome [23,37]. The rate of this complication among operative cases is unclear, but some long-term follow-up studies have shown that many patients do not develop this complication after 20 years following instrumentation with the Harrington rod [38].

Besides the flatback syndrome, other major issues bedeviled Harrington’s early efforts. To begin with, although the correction of the scoliotic deformity was achieved with instrumentation, most patients’ vertebral curvatures returned to their original magnitude within three to five months [7]. Attempts to address this by fixing more convex facets or incorporating both convex and concave facets proved unsuccessful. Harrington started experimenting with hooks and rods of different diameters. He developed thread-nut instrumentation with the goal of attaining dynamic spinal stabilization [24]. In addition, Harrington noticed that in many patients, the thread and nut loosened over time.

The subsequent evolution incorporated clamps and struts, the introduction of double-rod instrumentation for increased strength, and refining the hook and rod connection to prevent nut migration. Lastly, rod breakage was commonly seen in most patients intra- and post-operatively [27,28]. Harrington intended that the rod’s strength should precisely match the load requirements. He proposed that when instrumentation is applied correctly, the fusion of bone mass should have been achieved by the time the stainless steel rod fractures [14]. Attaining that ideal proved challenging, and in combination with other issues led to multiple revision surgeries in patients treated with spine instrumentation [14].

However, Harrington would go on to make several modifications to his instrumentation and technique over the years with the goal of improving outcomes [14,24,27,28]. Furthermore, newer approaches and instrumentation systems including the Luque implants and the Cotrel–Dubousset systems developed in response to these limitations [39].

## 6. Evolution to Long-Segment Spine Constructs

In the 1970s, the recognition of the imperative for a comprehensive approach to achieving the three-dimensional correction of spinal deformities spurred the emergence of innovative methodologies. These approaches built upon the foundational principles laid down by Harrington instrumentation. Furthermore, the introduction of segmental pedicle screws, a technique first described by the French spine surgeon Roy-Camille, paved the way for the development of systems aimed at the three-dimensional correction of spinal deformities [9].

One notably successful approach was the Cotrel–Dubousset system developed in the early 1980s. Adopting the use of segmental spinal instrumentation, this technique involves selective distraction and compression tailored to the specific degree of deformity at individual spinal levels [31]. The emphasis on segmental instrumentation contributed to more localized and customized corrections, ultimately optimizing spinal alignment and stability while comprehensively addressing deformities. The outcome was a modestly enhanced alignment not only in the sagittal and coronal planes but also in the axial plane. Early indications showed improved correction in the coronal plane with the Cotrel–Dubousset instrumentation.

The instrumentation sets of the Cotrel–Dubousset system included vertebral and sacral screws, as well as lamina and pedicle hooks. Over time, similar systems and devices, such as Isola, Moss Miami, and the Texas Scottish Rite implant system, among others, were introduced. It is worth noting that pedicle screws have remained relevant today as spine surgery evolved to the adoption of robotics and minimally invasive surgical techniques.

In contemporary spine surgery, significant progress has been made since the era of the Harrington instrumentation system. The materials used in modern devices have evolved beyond stainless steel to include more advanced options such as titanium and titanium alloys. These materials offer improved adaptability for conducive fracture healing and greater resistance to cyclic load and notch sensitivity [4].

It is noteworthy that pedicle screws remain a crucial component in today’s spine surgery landscape, particularly as the field has embraced robotics and minimally invasive techniques. Their enduring significance underscores their effectiveness in contributing to advancements in spinal surgical procedures, facilitating the adoption of more adaptive and patient-specific instrumentation.

Furthermore, there has been a substantial reduction in the rates of pseudoarthrodesis, thanks to advancements in fusion techniques. These include the incorporation of bone graft substitutes and biologics, which have proven to be effective in increasing fusion rates. Additionally, the integration of intra-operative neuromonitoring has become standard practice, enhancing the safety and precision of spinal surgeries. This collective progress signifies a paradigm shift towards more sophisticated and patient-tailored approaches in contemporary spine surgery.

## 7. Conclusions: Dr. Harrington’s Legacy 

Paul Harrington’s groundbreaking discoveries and innovations laid the foundation for fundamental biomechanical principles in modern spine surgery. The introduction of the Harrington rod represented a pivotal moment in managing spinal deformities, becoming the cornerstone for contemporary surgical techniques. Although replaced by more advanced systems by the late 1980s [9,30], the Harrington rod’s pioneering role remains unparalleled. The medical community’s dedication to refining approaches is evident in the shift towards long-segment spine constructs, addressing both clinical and biomechanical challenges.

Despite the advent of sophisticated instrumentation and techniques in spinal surgery, Paul Harrington’s contribution endures as a crucial chapter in its history. His innovative approach to treating scoliosis not only significantly influenced the evolution of spinal surgery but also played a pivotal role in improving patient outcomes.

The ongoing evolution of spinal surgery is driven by the synergistic relationship between clinical needs and technological advancements. As clinicians grapple with challenges in patient care, technology responds with innovative solutions, reshaping the landscape of spinal surgery. This dynamic interaction yields tangible benefits, including enhanced outcomes and reduced invasiveness, reflecting a commitment to advancing spinal surgery for the well-being of patients. The iterative process of identifying clinical needs and leveraging technological advancements underscores the continuous dedication to progress in spinal surgery.
